# A sting in the spit: widespread cross‐infection of multiple RNA viruses across wild and managed bees

**DOI:** 10.1111/1365-2656.12345

**Published:** 2015-03-03

**Authors:** Dino P. McMahon, Matthias A. Fürst, Jesicca Caspar, Panagiotis Theodorou, Mark J. F. Brown, Robert J. Paxton

**Affiliations:** ^1^ School of Biological SciencesMBCQueen's University BelfastBelfastBT9 7BLUK; ^2^ Institute of BiologyFree University BerlinSchwendenerstr. 114195BerlinGermany; ^3^ Department for Materials and EnvironmentBAM Federal Institute for Materials Research and TestingUnter den Eichen 8712205BerlinGermany; ^4^ School of Biological SciencesRoyal Holloway University of LondonEghamTW20 OEXUK; ^5^ IST Austria (Institute of Science and Technology Austria)3400KlosterneuburgAustria; ^6^ Institute for Biology, Martin‐Luther‐University Halle‐WittenbergHoher Weg 806120Halle (Saale)Germany; ^7^ German Centre for integrative Biodiversity Research (iDiv)Halle‐Jena‐Leipzig, Deutscher Platz 5e04103LeipzigGermany

**Keywords:** *Apis*, *Bombus*, decline, pathogen, spillover

## Abstract

1. Declining populations of bee pollinators are a cause of concern, with major repercussions for biodiversity loss and food security. RNA viruses associated with honeybees represent a potential threat to other insect pollinators, but the extent of this threat is poorly understood.
2. This study aims to attain a detailed understanding of the current and ongoing risk of emerging infectious disease (EID) transmission between managed and wild pollinator species across a wide range of RNA viruses.
3. Within a structured large‐scale national survey across 26 independent sites, we quantify the prevalence and pathogen loads of multiple RNA viruses in co‐occurring managed honeybee (*Apis mellifera*) and wild bumblebee (*Bombus* spp.) populations. We then construct models that compare virus prevalence between wild and managed pollinators.
4. Multiple RNA viruses associated with honeybees are widespread in sympatric wild bumblebee populations. Virus prevalence in honeybees is a significant predictor of virus prevalence in bumblebees, but we remain cautious in speculating over the principle direction of pathogen transmission. We demonstrate species‐specific differences in prevalence, indicating significant variation in disease susceptibility or tolerance. Pathogen loads within individual bumblebees may be high and in the case of at least one RNA virus, prevalence is higher in wild bumblebees than in managed honeybee populations.
5. Our findings indicate widespread transmission of RNA viruses between managed and wild bee pollinators, pointing to an interconnected network of potential disease pressures within and among pollinator species. In the context of the biodiversity crisis, our study emphasizes the importance of targeting a wide range of pathogens and defining host associations when considering potential drivers of population decline.

## Introduction

The ongoing biodiversity crisis threatens human health and global food security (Cardinale *et al*. 2012). Emerging infectious diseases (EIDs) have contributed significantly to species declines (Daszak, Cunningham & Hyatt 2000), with lethal chytridiomycosis in amphibians (Fisher, Garner & Walker 2009) and white‐nose syndrome in bats (Blehert *et al*. 2009) representing prominent recent examples. Infectious diseases may emerge through association with a host species (a ‘reservoir’) in which pathogens have become established, or where disease epidemiology may have recently changed due to perturbation (e.g. through arrival of a novel disease, or disease vector). The switching of pathogens between host species is a major cause of epidemics in humans and other vertebrate hosts (Woolhouse, Haydon & Antia 2005), and EIDs have potentially profound impacts on invertebrates providing important ecosystem services, which secure food production. However, the extent to which EIDs are an issue in invertebrates – and in insect pollinators particularly – is not clear.

Bees provide an essential ecosystem service in the form of crop pollination (Klein *et al*. 2007), but they are under pressure globally (Biesmeijer *et al*. 2006; Goulson, Lye & Darvill 2008; Brown & Paxton 2009; Williams & Osborne 2009; Potts *et al*. 2010; Vanbergen *et al*. 2013). Bumblebees are major wild pollinators in northern temperate climates (Goulson 2009), but they are declining in both the Old World (Williams 1982; Fitzpatrick *et al*. 2007; Kosior *et al*. 2007) and the New World (Bartomeus *et al*. 2013), with EIDs implicated as a cause of these declines (Cameron *et al*. 2011; Meeus *et al*. 2011; Fürst *et al*. 2014; Schmid‐Hempel *et al*. 2014). EIDs are known to be a major threat to the most widely used commercial pollinator, the honeybee (*Apis mellifera*), with the exotic ectoparasitic mite, *Varroa destructor*, meriting particular attention. The mite has risen to prominence due to its ability to act as a vector of several RNA viruses that previously persisted relatively benignly in honeybee colonies, most notably deformed wing virus (DWV), but also viruses belonging to the acute bee paralysis virus (ABPV) complex (Genersch & Aubert 2010) and slow bee paralysis virus (SBPV) (Carreck, Ball & Martin 2010; Santillán‐Galicia *et al*. 2014). In the case of DWV, the arrival of *V. destructor* has been directly linked to increased prevalence and virus loads in honeybees (Martin *et al*. 2012).

Wild pollinators harbour pathogens previously associated with honeybees (Genersch *et al*. 2006; Singh *et al*. 2010; Peng *et al*. 2011; Evison *et al*. 2012; Graystock *et al*. 2013; Levitt *et al*. 2013; Ravoet *et al*. 2014), and for at least one emerging RNA virus, disease in managed honeybees and wild bumblebees is linked (Fürst *et al*. 2014). The association of pathogens with managed honeybees is in part a reflection of study bias, but the trend may also point to an emerging problem of infectious RNA viruses in wild bees – triggered, perhaps, by the arrival of *V. destructor* mites in the western honeybee some 40 years ago (Rosenkranz, Aumeier & Ziegelmann 2010). For the great majority of RNA viruses in wild bees, detailed knowledge of prevalence and level of infection (pathogen load) is still lacking. This represents a significant gap in understanding, particularly given the prominent role that RNA virus diseases are believed to play in causing managed honeybee colony loss (Schroeder & Martin 2012).

We therefore conducted a comprehensive field analysis of honeybee and wild bumblebee populations across Great Britain and the Isle of Man to (i) understand the contemporary landscape prevalence of common RNA viruses thought to be associated with honeybees, (ii) quantify and compare the individual infection levels of RNA viruses in bee foragers, (iii) assess the extent to which RNA virus spillover is occurring between honeybees and bumblebees (in either direction). We show that multiple RNA viruses are prevalent in wild bee populations and present evidence for recent and widespread circulation of viral diseases between Britain's primary managed and wild bee pollinators.

## Materials and methods

### Field Sampling and RNA Extraction

Field sampling methodology and RNA extraction follows Fürst *et al*. (2014). Briefly, we collected free flying honeybees and bumblebees from flowers at 26 sites (A‐Z) across Great Britain and the Isle of Man, each separated by at least 30 km (mean ± SD distance in km = 69·21 ± 26·39). The collection area covered at least 1000 m^2^ at each location, and where possible, all bees were collected within a single day. Time taken (in minutes) to collect 20 *A. mellifera* and 20 *Bombus* spp. individuals was recorded as an estimate of abundance. Honeybees and up to four species of bumblebees from each site were then screened for the presence and quantity of a range of viruses. Honeybee or bumblebee abdomens were bisected longitudinally, one‐half of which was submerged in RLT buffer and disrupted in a Tissue lyser II (Qiagen, Manchester, UK) at 30 Hz for 2 min followed by 20 Hz for 2 min prior to RNA isolation. Total RNA was extracted manually using the RNeasy mini kit (Qiagen, Manchester, UK) following manufacturer's instructions.

### Pathogen Detection

We screened for a wide range of known positive‐sense single‐stranded RNA viruses, by employing multiple ligation‐dependent probe amplification (MLPA) using the RT‐MLPA^®^ kit (MRC‐Holland, Amsterdam, Netherlands). We used probes designed for the positive strand of the following six composite positive‐sense single‐stranded RNA virus targets (De Smet *et al*. 2012): (i) black queen cell virus (BQCV); (ii) deformed wing virus, *Varroa destructor* virus and kakugo virus (DWV/KV/VDV‐1); (iii) acute bee paralysis virus, Israeli acute paralysis virus and Kashmir bee virus (ABPV/IAPV/KBV); (iv) slow bee paralysis virus (SBPV); (v) sac brood virus (SBV); and (vi) chronic bee paralysis virus (CBPV) and, as a housekeeping (control) gene, β‐actin. Notable viruses such as the Lake Sinai viruses (LSV 1 and 2) have been recorded in North America (Runckel *et al*. 2011) and Europe (Granberg *et al*. 2013). While these were not included in the current study, we acknowledge that they may also be transmitted across species. Amplified fragments were resolved by capillary electrophoresis on a QIAxcel (Qiagen, Hilden, Germany), using a positive virus acceptance threshold of 0·1 relative fluorescence units. Samples were excluded from further analysis if the housekeeping gene, β‐actin, fell below this threshold.

For each MLPA positive virus target, samples were analysed by qRT‐PCR to identify the specific virus (in the case of DWV/KV/VDV‐1 and ABPV/IAPV/KBV) and to estimate individual viral load. This allowed us to differentiate between VDV‐1 and DWV/KV and between ABPV, IAPV and KBV. KV and DWV are very closely related and were not differentiated by qRT‐PCR. Total cDNA was synthesized using M‐MLV Revertase (Promega, Mannheim, Germany) following manufacturer's instructions, using 500 ng of sample RNA. For absolute quantification, duplicate qRT‐PCR was performed for each sample with a Bio‐Rad C1000, using SYBRgreen Sensimix (Bioline, Luckenwalde, Germany) in the following program: 5 min at 95 °C, followed by 40 cycles of 10 s at 95 °C, 30 s at 57 °C and 30 s at 72 °C (read). Duplicate β‐actin reactions were also amplified for all samples as an internal reference marker. A negative control containing RNA‐free HPLC water and a virus‐positive sample were included as controls in each reaction run. To account for potential variation in sample quality, an upper cycle threshold (*Ct*) of 35 was set for β‐actin, above which samples were not included in quantitative analysis. Given the previous positive detection of virus by MLPA, an upper threshold for virus quantification by qRT‐PCR was not applied. We used specific primers for the following viruses: BQCV; DWV; VDV‐1; ABPV; IAPV; KBV; SBPV; SBV (see Table S1, Supporting information). Following PCR, DNA was denatured for 1 min at 95 °C and cooled to 55 °C for 1 min. A melting profile was generated from 55 to 95 °C (0·5 °C per second increments). Quantification was calculated using duplicate DNA standard curves of purified flanking PCR products (DWV, VDV‐1, Table S1, Supporting information for primers) or plasmids (BQCV; ABPV; SBPV), with efficiencies of 98·4% (DWV), 99·9% (VDV‐1), 96·2% (BQCV), 101·3% (ABPV) and 93·1% (SBPV), and correlation coefficients (*R* ^2^) from 0·995 to 0·999.

### Sequencing

To confirm the identity of viruses, we cloned and sequenced virus fragments from single honeybees and up to two bumblebees that contained high levels of BQCV, ABPV or SBPV. qRT‐PCR products were purified using the Qiaquick PCR Purification Kit (Qiagen, Hilden, Germany) and cloned directly using the pGEM T Easy Vector system (Promega, Mannheim, Germany) following manufacturer's instructions. Plasmid DNA was isolated using a Spin Miniprep Kit (Qiagen, Hilden, Germany). Up to five clones per sample were sequenced in forward and reverse orientation (GATC Biotech, Constance, Germany), and aligned by eye to genome references of BQCV (NC_003784), ABPV (NC_002548) and SBPV (NC_014137). DWV and VDV‐1 sequences have been analysed previously (Fürst *et al*. 2014).

### Statistical Analysis

Analyses were performed in r v 3.0.2 (R Core Team 2013). RNA virus prevalence differences between pollinator genera were compared in a test of proportions (χ^2^ test), using a Bonferroni correction (α = 0·003; six species; 15 multiple comparisons) for comparisons between species. So that differences among species of different samples sizes could be meaningfully compared, we estimated true prevalence and 95% confidence intervals using the r library ‘epir’ v0.9‐54, with sensitivity and specificity both set at 95%. Disease prevalence was mapped to sites using the ‘mapplots’ package v1.4, or estimated using Gaussian kernel estimators using the package ‘prevr’ as described previously (Fürst *et al*. 2014). Distributions of infectious loads were compared using Kolmogorov–Smirnov tests.

To explore possible drivers of RNA virus prevalence in managed and wild bees, we performed generalized linear mixed models (GLMM) with binomial error structure using the package ‘lme4’ v.1.0‐6. Prior to any statistical analysis, we used Moran's I and spline correlograms (package ‘ade4’ v1.6‐2: Dray & Dufour 2007; package ‘ncf’ v1.1‐5: Bjørnstad 2013) to test for potential spatial autocorrelation. The geographical distance between all pairs of sites was calculated, and results indicated there was no significant spatial autocorrelation for any of the RNA viruses in *A. mellifera* or *Bombus* spp. (*P* > 0·05). *V. destructor* mites have caused both an increase in viral load and prevalence of several RNA viruses in western honeybee populations*,* including DWV (Martin *et al*. 2012), ABPV (Genersch & Aubert 2010) and SBPV (Carreck, Ball & Martin 2010; Santillán‐Galicia *et al*. 2014). We hypothesize that the association of these viruses with *A. mellifera* has resulted in disease spillover into wild *Bombus* spp. populations. We therefore modelled *Bombus* virus prevalence as dependent on *A. mellifera* virus prevalence, *A. mellifera* abundance, *Bombus* abundance, latitude, longitude and landcover type, while treating site and species as random effects. However, to account for uncertainty surrounding the true directionality of pathogen spillover, we also conducted models with *A. mellifera* virus prevalence as the response variable, retaining all other predictors except species as a random effect. We conducted separate GLMMs for BQCV, DWV and ABPV. SBV and SBPV were not modelled due to insufficient positive samples (*n* = 4 *Bombus* and *n* = 5 *A. mellifera* individuals, respectively). Site G was removed prior to statistical analysis as no *A. mellifera* foragers were collected at this site. *A. mellifera* and *Bombus* spp. abundance were log‐transformed, and all quantitative predictors were standardized to a mean of zero and standard deviation of one prior to analysis. Models were simplified by backward stepwise selection based on AIC (‘drop1’ function). We used variance inflation factors (VIF) to check for colinearity among our explanatory variables, applying a cut‐off value of 3. Variables with a high VIF were removed one at a time until all VIF values were below 3 (Zuur *et al*. 2009). Both conditional (*r* ^2^_c_, all factors) and marginal (*r* ^2^_*m*_, fixed factors only) values are shown.

In addition to individual GLMMs, we summed the prevalence of each virus at each site and modelled the resulting total virus prevalence data in a general linear model (GLM) to explore the overall relationship of virus prevalence between *A. mellifera* and *Bombus* spp. We modelled *Bombus* prevalence as dependent on *A. mellifera* virus prevalence, *A. mellifera* abundance, *Bombus* abundance, latitude, longitude and landcover type. As before, abundance was log‐transformed and quantitative predictors were standardized. As before, we also conducted a GLM with *A. mellifera* virus prevalence as the response variable. Model selection was performed using an automatic approach (package ‘glmulti’, Calcagno & De Mazancourt 2010) using the AICc method. Nagelkerke *r* ^*2*^ values are shown.

## Results

### Data Summary and Virus Composition

Of 792 sampled bees, the following passed β‐actin quality control (for sample sizes, collection times and species composition by site, see Table S2, Supporting information): 92% *A. mellifera* (*n* = 237); 100% *B. hortorum* (*n* = 30); 100% *B. jonellus* (*n* = 1); 90% *B. lapidarius* (*n* = 169); 93% *B. lucorum* (*n* = 89); 90% *B. pascuorum* (*n* = 55); 100% *B. monticola* (*n* = 7); 100% *B. pratorum* (*n* = 3); and 64% *B. terrestris* (*n* = 92). *B. terrestris* β‐actin was identified to contain a ligation‐site sequence mismatch, and the proportion of samples passing quality control was lower for this species. In future cross‐species comparisons, MLPA probes should be designed for a wider range of housekeeping genes from which uniform markers across bee species can be selected.

For both *A. mellifera* and *Bombus* spp., we detected VDV‐1 and DWV/KV from the DWV/VDV‐1/KV complex, but only ABPV from the ABPV/IAPV/KBV complex. DWV complex strains are closely related at proteolytic sites (de Miranda & Genersch 2010) and naturally recombine (Moore *et al*. 2011). We therefore refer to the DWV complex as ‘DWV’ from hereon. Although ABPV/IAPV/KBV are thought to be distinct viruses, we also refer to the ABPV complex as ‘ABPV’ from hereon, due to the inability to detect either IAPV or KBV in any sample (*n* = 54 individuals were positively detected in MLPA, of which 47 were positive for ABPV, but none were positive for IAPV or KBV. Samples that were negative for all three qPCR targets (*n* = 7) could be attributable to qRT‐PCR primer mismatches preventing amplification). Analysis of nucleotide sequences further confirmed the sequence identity of BQCV, ABPV and SBPV in infected *A. mellifera* and *Bombus* spp. foragers (Fig. S1, Supporting information). Unrooted trees for SBPV and BQCV sequences are given in Fig. S2 (Supporting information) (ABPV is not displayed as all sequences were identical). SBPV clones from *B. pascuorum* and *A. mellifera* are similar or identical, whereas *B. terrestris* is represented by two diverging haplotypes. For BQCV, clones from each species were more clearly separated, but this is unsurprising given that each individual host bee originated from a different site. Interestingly, *B. terrestris* was again represented by two distinct haplotypes.

Both MLPA and qRT‐PCR assays did not specifically amplify the negative strand of RNA viruses, and as such, they did not test for actively replicating virus directly. Nevertheless, our methods provide a reliable indicator as to the presence and potential severity of viral infections in bee foragers by employing a multiplexed presence/absence screen followed by quantification.

### Prevalence

In an analysis combining all RNA viruses as a single response, the true prevalence was 51% (95% CI: 44%, 58%) in *A. mellifera* and 23% (95% CI: 19–27%) in *Bombus* spp. (χ^2^_1 _= 50·0, *P* < 0·0001). Most viruses occurred singly, with co‐occurrence of two and three viruses being detected in, respectively, 7% (95% CI: 4–12%) and 1% (95% CI: 0–3%) of *A. mellifera* individuals, and 3% (95% CI: 2–5%) and 0·2% (95% CI: 0–1%) of *Bombus* spp. individuals (Fig. S3, Supporting information). The proportion of coinfected individuals did not depart from null expectations (*A. mellifera*: χ^2^_3 _= 0·5, *P* = 0·918; *Bombus*: χ^2^_3 _= 4·8, *P* = 0·189). The most prevalent virus was DWV in honeybees (36%, 95% CI: 30–43%) and ABPV in bumblebees (11%, 95% CI: 8–14%; Table 1). CBPV was not recorded from any sample.

**Table 1:** Virus prevalence in per cent for six virus targets, with 95% CI in square brackets. Sample numbers for each pollinator genus are shown in parentheses

Pollinator	BQCV	DWV	ABPV	SBPV	SBV	CBPV
*A. mellifera* (237)	15 [10, 20]^a^	36 [30, 43]^a^	5 [2, 9]	2 [1, 5]	2 [1, 4]	0 [0, 2]
*Bombus* spp. (453)	6 [4, 8]	3 [2, 5]	11 [8, 14]^a^	5 [3, 7]	1 [0, 2]	0 [0, 1]

a Significantly higher virus prevalence in a test of proportions (*A. mellifera* vs. *Bombus* spp.).

**Figure 1:**
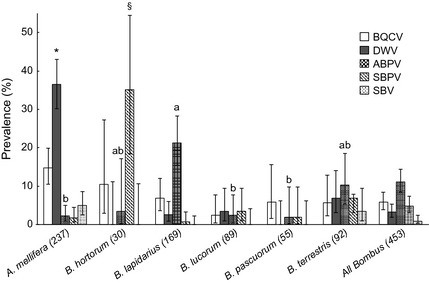
Prevalence of each virus mapped by individual species, showing mean true estimates and 95% CIs. Bonferroni‐corrected chi‐square test for multiple comparisons: *DWV in *Apis mellifera* significantly higher prevalence compared with all other species. ^§^ SBPV in *Bombus hortorum* significantly higher prevalence compared with all other species. ABPV: significant multiple comparisons indicated by letters a and b. Note that ‘all *Bombus’* is not included in statistical comparison (see Table 1).

Five RNA virus targets were detected in both *A. mellifera* and *Bombus* spp. (Table 1). In a test of proportions, BQCV (χ^2^_1 _= 13·2, *P* < 0·001) and DWV (χ^2^_1 _= 126·4, *P* < 0·0001) were more prevalent in *A. mellifera*, whereas ABPV was more prevalent in *Bombus* spp. (χ^2^_1 _= 6·3, *P* < 0·05). Although SBPV and SBV were more prevalent in *Bombus* spp. and *A. mellifera,* respectively, differences between host genera were not statistically significant (χ^2^_1 _= 2·1, *P* = 0·15; χ^2^_1 _= 0·32, *P* = 0·57, for SBPV and SBV, respectively). In a comparison of virus prevalence among the five commonest host species (*n* > 10 collected individuals), we found that DWV and SBPV were significantly more prevalent in *A. mellifera* and *B. hortorum*, respectively (Fig. 1). ABPV also occurred at significantly higher prevalence in *B. lapidarius* compared with *A. mellifera*,* B. lucorum* and *B. pascuorum,* but not *B. hortorum* or *B. terrestris* (Fig. 1).

**Figure 2:**
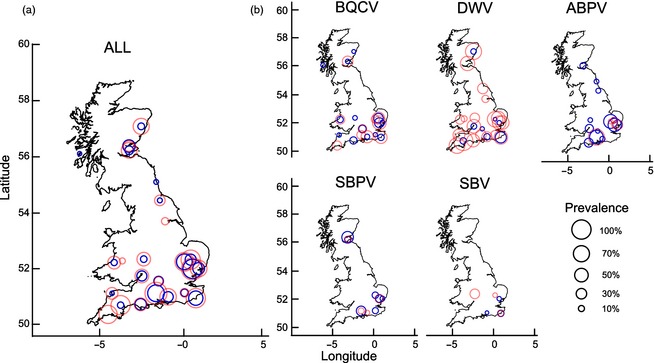
Prevalence of (a) combined and (b) individual RNA viruses mapped by site and pollinator genus (%). *Apis mellifera* and *Bombus* spp. are represented as light red and dark blue circles, respectively.

**Figure 3:**
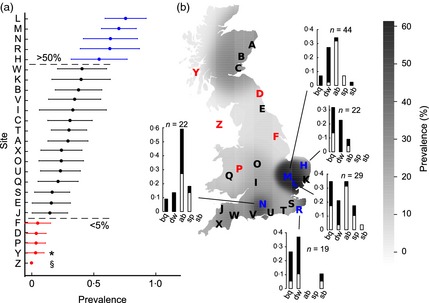
(a) Combined *Apis mellifera* and *Bombus* spp. virus prevalence by site. Mean and 95% CIs calculated from presence/absence individual data (single and multiple infections treated equally). Sites with ≤5% and >50% RNA virus prevalence are emphasized (red and blue, respectively). *island of Colonsay (site Y) and ^§^Isle of Man (site Z). (b) RNA virus prevalence mapped by Gaussian kernel estimation, with site locations overlaid. Bar graphs summarize the prevalence of individual viruses at blue sites, with proportions derived from *Bombus* spp (white) and *A. mellifera* (black) indicated. bq = BQCV; dw = DWV; ab = ABPV; sp = SBPV; sb = SBV.

We mapped the prevalence of both individual and combined RNA viruses by site and pollinator genus (Fig. 2). These indicated that disease prevalence between managed (*A. mellifera*) and wild (*Bombus* spp.) bees were linked. In GLMMs of individual viruses, we found that prevalence of BQCV (final model *r* ^2^_*c* _= 0·28; *r* ^2^_*m* _= 0·28), ABPV (final model *r* ^2^_*c *_= 0·51; *r* ^2^_*m *_= 0·08) and DWV (final model *r* ^2^_*c *_= 0·39; *r* ^2^_*m *_= 0·12) in *A. mellifera* had a positive effect on BQCV, ABPV and DWV prevalence in *Bombus* spp., respectively (Table 2, Fig. S4, Supporting information), although this effect was marginally not significant in the final DWV model. Additionally, abundance of *Bombus* spp. and *A. mellifera* had a negative and positive effect, respectively, on BQCV prevalence in *Bombus* spp. When we reconstructed models with *A. mellifera* virus prevalence as dependent on *Bombus* spp. pathogen prevalence, both the final models and significant predictors closely matched the original models that treated *Bombus* spp. prevalence as the response variable (Table S3, Supporting information). In a GLM of combined RNA viruses (where prevalence data were summed for all five positively detected RNA viruses), we found that disease prevalence in *A. mellifera* also had an overall positive effect on disease prevalence in *Bombus* spp. (final model Nagelkerke *r* ^2^ **= 0·62, Table 2, Fig. S4, Supporting information), and that latitude was also a significant predictor. Again, when we reconstructed the GLM with *A. mellifera* virus prevalence as dependent on *Bombus* spp., the final models matched the original GLM (Table S3, Supporting information). Sites harbouring highest overall RNA virus prevalence were concentrated in SE England. On the other hand, those harbouring lowest RNA virus prevalence were located in remote western regions, including two *V. destructor* mite‐free islands (Y: island of Colonsay; Z: Isle of Man) that contained the lowest overall disease prevalence across all sites (Fig. 3).

**Table 2:** (a) Best model explaining individual virus prevalence in *Bombus* spp. using GLMMs and AIC for model selection. Note that the sign of the parameter estimates for abundance is opposite to the direction of the relationship between variables due to the way in which abundance was measured (see Materials and methods). (b) Best model explaining total RNA virus prevalence in *Bombus* spp., using a GLM and AICc for model selection

Response (Model)	Virus	Parameters	Estimate	SE	*z*‐value	*P*‐value
(a)	BQCV	Intercept	−3·212	0·305	−10·542	
		*Apis* BQCV	0·542	0·186	2·917	0·004^a^
		*Apis* abundance	−0·686	0·335	−2·046	0·041^a^
*Bombus* virus prevalence		*Bombus* abundance	0·813	0·345	2·356	0·018^a^
(GLMM)	DWV	Intercept	−4·185	0·506	−8·275	0·055
		*Apis* DWV	0·818	0·426	1·918	
	ABPV	Intercept	−3·725	0·586	−6·356	0·008^a^
		*Apis* ABPV	0·727	0·274	2·654	
(b)	ALL	Intercept	0·050	0·063	0·792	
*Bombus* virus prevalence		*Apis* all viruses	0·367	0·097	3·777	0·001^a^
(GLM)		Longitude	0·091	0·039	2·350	0·028^a^

a Significant variables.

### Virus Load

**Figure 4:**
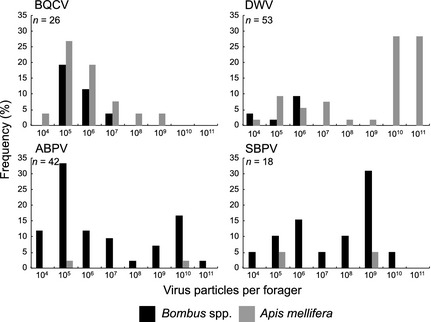
Comparison of relative frequencies (%) of inferred absolute virus loads in *Apis mellifera* and *Bombus* spp. individual foragers.

We quantified BQCV, DWV, ABPV, SBPV and SBV from the positively detected *A. mellifera* and *Bombus* spp. foragers (Fig. 4). For BQCV, virus loads between *A. mellifera* and *Bombus* spp. were not significantly different (two‐sided Kolmogorov–Smirnov, *D*_9,17_ *= *0·38, *P* = 0·63), with putatively low‐level infections (10^4^–10^6^ virus particles) predominating in bees from both genera. For DWV, viral loads in *A. mellifera* foragers were greater than in *Bombus* spp. (one‐sided Kolmogorov–Smirnov, *D*_8,45_ *= *0·63, *P* < 0·05), consistent with the presence of high‐level infections in *A. mellifera* (10^10^–10^11^ virus particles) vs. low‐level infections in *Bombus* spp. (10^4^–10^6^ virus particles). We detected a wide range of ABPV and SBPV virus loads in *Bombus* spp. (10^4^–10^11^ ABPV and 10^5^–10^11^ SBPV particles), but sample sizes were inadequate in *A. mellifera* (ABPV and SBPV: *n* = 2 each) to be able to compare distributions between pollinator genera. SBV was not detected in any of the samples by qRT‐PCR (*n* = 8 individuals positively detected by MLPA).

## Discussion

In a comprehensive field survey of managed and wild bee pollinators, we found that common RNA viruses previously associated with honeybees are widespread in bumblebee populations, and that viruses vary substantially in terms of individual pathogen load and population‐level prevalence. Significantly, we demonstrate a positive association in disease prevalence between managed and wild bees, indicating that disease spillover may be an important general aspect of RNA virus epidemiology in bee pollinators.

### RNA Viruses are Widespread in Wild Bees

RNA viruses are prevalent in wild bee populations and occur broadly in the landscape. More than one in every five bumblebee foragers sampled contained at least one of the RNA viruses that were screened for. We note that the total impact of RNA viruses on wild bees is likely to be higher than suggested from our prevalence data, as severely affected individuals may be less likely to fly and forage.

Of the targeted viruses, we found that BQCV, DWV, ABPV and SBPV occurred commonly in bumblebee foragers. Previous studies have indicated that wild bees could harbour RNA viruses typically associated with honeybees (Genersch *et al*. 2006; Singh *et al*. 2010; Evison *et al*. 2012), and in the case of DWV, that spillover is likely occurring between managed and wild bee populations (Fürst *et al*. 2014). By combining a structured survey of multiple RNA viruses with a quantitative analysis of pathogen load, we show that not only DWV but also BQCV is widespread in wild bee populations, and that bumblebee foragers largely harbour low levels of these viruses. In honeybee foragers, we find that BQCV occurs at similarly low levels, but that levels of DWV are significantly higher, as would be expected given the prominent role of *V. destructor* mites in vectoring this virus. Surprisingly, we find that ABPV and SBPV are more prevalent in bumblebee than honeybee foragers (although this difference is not significant for SBPV), and that bumblebees harbour a wide range of pathogen loads, including a substantial proportion of putatively high infections (>10^9^ virus particles per individual).

ABPV and SBPV were significantly more prevalent in *B. lapidarius* and *B. hortorum,* respectively, as compared with most other bee species (including *A. mellifera*), suggesting that differences in host susceptibility or quality may exist (Ruiz‐González *et al*. 2012). *B. lapidarius* is common in England and Wales, while *B. hortorum* is widespread across Great Britain (Goulson 2009), so there is no clear pattern of pathogen prevalence and bumblebee rarity among our samples. Alternatively, this could indicate that species such as *B. lapidarius* and *B. hortorum* are simply more tolerant to infection than others. In addition to factors relating to host immunity, life history parameters linked to phenology, such as the relative abundance of bumblebee foragers and/or reproductives relative to honeybees (the viral titres of which are known to vary temporally, Runckel *et al*. 2011) may play an important role in between‐species differences in disease prevalence and transmission. Additionally, although *V. destructor* mites are able to vector both ABPV and SBPV in honeybees, these viruses were found at lower prevalence in honeybee foragers. In the case of ABPV, while there is evidence that the arrival of *V. destructor* mites has increased the prevalence of ABPV in *A. mellifera* (reviewed in Genersch & Aubert 2010), the reduced survival of infected pupae could explain why ABPV is detected less frequently than DWV in honeybee foragers (Sumpter & Martin 2004; Schroeder & Martin 2012).

The situation for SBPV is less clear, although both field (Carreck, Ball & Martin 2010) and laboratory experiments (Santillán‐Galicia *et al*. 2014) suggest that it may be transmitted between honeybees via *V. destructor* mites and that it may be more virulent than DWV. With respect to wild bees, and bumblebees in particular, virtually nothing is known of the epidemiology of these RNA virus. Furthermore, controlled infection experiments are required to improve basic knowledge of the impacts of these and other RNA virus in non‐*Apis* bees (e.g. Meeus *et al*. 2014), and to test competing hypotheses for host species differences in disease prevalence.

### Circulation of Viruses between Managed and Wild Bees

We detected a significant association between prevalence of viruses in honeybees and bumblebees, both in a combined RNA virus analysis, and for viruses analysed separately, notably BQCV and ABPV. Interestingly, BQCV prevalence in bumblebees was associated negatively with *Bombus* abundance but positively with *A. mellifera* abundance. This might indicate that lower bumblebee abundance is caused by higher BQCV prevalence, itself the result of higher honeybee abundance. However, directions of causality remain equivocal, and given current understanding, we advocate restraint in the extent to which our models are interpreted. Our data also indicated an association between pollinators for DWV, but the relationship was not as strong as the effect detected in a previous study (Fürst *et al*. 2014). Several factors could be responsible for this variation. First, Fürst *et al*. (2014) based their analysis on a data set comprised of a random subsample of 10 individuals of the two commonest species, whereas in this study, we included every successfully amplified sample at each site regardless of species. Secondly, many individuals were differentially excluded based on separate quality control measures, which resulted in substantially reduced representation of *B. terrestris* in our study. Finally, the sensitivity of MLPA, a multiplexed approach based on competitive PCR, is lower than a single RT‐PCR approach, resulting in a higher likelihood of false negatives (de Miranda *et al*. 2013). Given the last consideration, it is probable that the impact of RNA viruses on bee populations is greater than we report.

Several outstanding questions emerge from our findings that merit further attention, given the pressures faced by bumblebees globally (Fitzpatrick *et al*. 2007; Bartomeus *et al*. 2013) and the potential role of pathogens in these declines (Cameron *et al*. 2011; Meeus *et al*. 2011). First, the direction of disease spillover between managed and wild bees represents a major unanswered question. By extension, whether honeybee or bumblebee populations are more important as natural reservoirs of RNA virus infections also remains unclear. Based on prevalence alone, BQCV and DWV appear more closely linked with honeybees whereas ABPV and SBPV are more common in bumblebees. Unfortunately, prevalence levels taken at a single time point are not informative with regard to understanding the primary reservoir host(s), or by extension, the principle direction of disease transmission. Spillover may be bidirectional, with both pollinator genera functioning as suitable long‐term reservoirs (although the perennial life cycle and presence of *V. destructor* mites may favour the honeybee as a more likely long‐term disease source). We also cannot exclude the possibility that one or more unknown species from the wider invertebrate community is the primary disease reservoir. Equally, the main reservoir may be a low‐prevalence host, whereupon entering a second host the virus spreads rapidly, resulting in an epidemic and higher observed prevalence. For example, it is plausible that increased ABPV prevalence in bumblebees is due indirectly to the increased exposure to infectious virus particles emerging from *V. destructor*‐infested honeybee colonies that contain higher than normal loads of ABPV (Genersch & Aubert 2010). Interestingly, we found that the sites least affected by disease in this study are also those where *V. destructor* has not yet become established in honeybees, although these are also the most remote island locations.

Our study significantly extends previous findings that suggested horizontal transmission of pathogens between bee pollinators. However, demonstration of the mechanistic basis of host switching in the field is still lacking. Potential transmission routes include direct contacts between bees (bumblebees entering managed honeybee colonies are not uncommon, for example, Genersch *et al*. 2006), or more likely, via indirect interactions such as through shared use of floral resources (McArt *et al*. 2014). Unfortunately, the field is largely devoid of observational or experimental data that tackle the issue of pathogen transmission at flowers directly (although see Durrer & Schmid‐Hempel 1994).

Singh *et al*. (2010) report that pollen pellets carried by honeybee foragers (in addition to stored honey) contain viable RNA virus, despite absence of virus in the forager itself. This suggests that infective inocula reside at flowers and may be collected by flower visitors. However, the probabilities of infectious material being deposited and subsequently acquired by a new host while remaining viable are unknown. As outlined previously (McArt *et al*. 2014), we expect traits such as flower complexity (Durrer & Schmid‐Hempel 1994), pollinator flower‐handling time and floral secondary compounds [e.g. antimicrobial compounds, host immune modulators (Mao, Schuler & Berenbaum 2013)] to influence the likelihood of infection. Pathogen transmission at flowers remains poorly understood, but bridging this gap in understanding should be a priority for pollinator research.

### Emerging Environmental Pressures on Wild Bees

EIDs represent one of several stressors that have been implicated in bee pollinator declines. Other major drivers are thought to include habitat change and loss (reviewed in Potts *et al*. 2010), and more recently, pesticides (Desneux, Decourtye & Delpuech 2007; Gill, Ramos‐Rodriguez & Raine 2012; Whitehorn *et al*. 2012; Williamson & Wright 2013). However, an explicit causal link between any single factor and bee declines has not emerged (Vanbergen *et al*. 2013). It is plausible that several factors acting in synergy serve to amplify pressures on pollinators (González‐Varo *et al*. 2013), or that a range of different factors may produce similar levels of stress at the colony level (Bryden *et al*. 2013).

Among the bumblebees in this study, we found a significant proportion of the active foraging workforce (>20%) to harbour RNA viruses, often at putatively low levels. In combination with other stressors, such pervasive disease pressures could have a general and profound impact on the long‐term health of bee populations. Recent studies have reported interactions between sublethal doses of neonicotinoid pesticides and pathogens, with significant impacts on virus replication and host immunity (Di Prisco *et al*. 2013) and bee mortality (Doublet *et al*. 2014). Alongside the lethal impacts of environmental stressors (either acting in isolation or in combination), the role of sublethal chronic stress has also attracted attention. Both pathogens (Mayack & Naug 2009) and pesticides (Gill, Ramos‐Rodriguez & Raine 2012) can act as chronic stressors, with negative impacts on social bee colony function, leading eventually to colony failure (Bryden *et al*. 2013).

Our findings reveal the widespread prevalence in wild bee populations of multiple RNA viruses previously associated with honeybees. We present evidence of ongoing or recent transmission of viral diseases between managed and wild bee populations, but we remain cautious in speculating on the main direction of spread between pollinator genera, or in making predictions about which bee species act as the principle reservoir for infectious disease. The arrival of *V. destructor* mites heralded a major shift in the epidemiology of several RNA viruses in the western honeybee, with potentially wide implications for disease spillover among wild pollinators. While we show that RNA viruses are widespread in wild bees, it is unclear to what extent viral challenge impacts bumblebees at the population level, either in isolation or in combination with other stressors.

## Supporting information


**Table S1.** List of qRT‐PCR primers used in this study

**Table S2.** Detailed sample information by collection site

**Table S3.** GLMM and GLM final models with *A. mellifera* virus prevalence as the response variable

**Fig. S1.** BQCV, ABPV and SBPV cloned sequence alignments.

**Fig. S2.** Phylogenetic trees of cloned BQCV and SBPV sequences.

**Fig. S3.** Summary prevalence (%) of RNA viruses in *A. mellifera* and *Bombus* spp.

**Fig. S4.** Linear regressions of raw *A. mellifera* and *Bombus* spp. RNA virus prevalence data.

## References

[jane12345-bib-0001] Bartomeus, I. , Ascher, J.S. , Gibbs, J. , Danforth, B.N. , Wagner, D.L. , Hedtke, S.M. *et al* (2013) Historical changes in northeastern US bee pollinators related to shared ecological traits. Proceedings of the National Academy of Sciences of the United States of America, 110, 4656–4660. 23487768 10.1073/pnas.1218503110 PMC3606985

[jane12345-bib-0002] Biesmeijer, J.C. , Roberts, S.P.M. , Reemer, M. , Ohlemuller, R. , Edwards, M. , Peeters, T. *et al* (2006) Parallel declines in pollinators and insect‐pollinated plants in Britain and the Netherlands. Science, 313, 351–354. 16857940 10.1126/science.1127863

[jane12345-bib-0003] Bjørnstad, O.N. (2013) ncf: Spatial nonparametric covariance functions. R package version 1.1‐5.

[jane12345-bib-0004] Blehert, D.S. , Hicks, A.C. , Behr, M. , Meteyer, C.U. , Berlowski‐Zier, B.M. , Buckles, E.L. *et al* (2009) Bat white‐nose syndrome: an emerging fungal pathogen? Science, 323, 227. 18974316 10.1126/science.1163874

[jane12345-bib-0005] Brown, M.J.F. & Paxton, R.J. (2009) The conservation of bees: a global perspective. Apidologie, 40, 410–416.

[jane12345-bib-0006] Bryden, J. , Gill, R.J. , Mitton, R.A.A. , Raine, N.E. & Jansen, V.A.A. (2013) Chronic sublethal stress causes bee colony failure. Ecology Letters, 16, 1463–1469. 24112478 10.1111/ele.12188 PMC4299506

[jane12345-bib-0007] Calcagno, V. & De Mazancourt, C. (2010) Glmulti: an R package for easy automated model selection with (generalized) linear models. Journal of Statistical Software, 34, 1–29.

[jane12345-bib-0008] Cameron, S.A. , Lozier, J.S. , Strange, J.P. , Koch, J.B. , Cordes, N. , Solter, L.F. *et al* (2011) Patterns of widespread decline in North American bumble bees. Proceedings of the National Academy of Sciences of the United States of America, 108, 662–667. 21199943 10.1073/pnas.1014743108 PMC3021065

[jane12345-bib-0009] Cardinale, B.J. , Duffy, J.E. , Gonzalez, A. , Hooper, D.U. , Perrings, C. , Venail, P. *et al* (2012) Biodiversity loss and its impact on humanity. Nature, 486, 59–67. 22678280 10.1038/nature11148

[jane12345-bib-0010] Carreck, N.L. , Ball, B.V. & Martin, S.J. (2010) Honey bee colony collapse and changes in viral prevalence associated with *Varroa destructor* . Journal of Apicultural Research, 49, 93–94.

[jane12345-bib-0011] Daszak, P. , Cunningham, A.A. & Hyatt, A.D. (2000) Emerging infectious diseases of wildlife threats to biodiversity and human health. Science, 287, 443–449. 10642539 10.1126/science.287.5452.443

[jane12345-bib-0012] De Smet, L. , Ravoet, J. , de Miranda, J.R. , Wenseleers, T. , Mueller, M.Y. , Moritz, R.F. *et al* (2012) BeeDoctor, a versatile MLPA‐based diagnostic tool for screening bee viruses. PLoS ONE, 7, e47953. 23144717 10.1371/journal.pone.0047953 PMC3483297

[jane12345-bib-0013] Desneux, N. , Decourtye, A. & Delpuech, J.M. (2007) The sublethal effects of pesticides on beneficial arthropods. Annual Review of Entomology, 52, 81–106. 10.1146/annurev.ento.52.110405.091440 16842032

[jane12345-bib-0014] Di Prisco, G. , Cavaliere, V. , Annoscia, D. , Varricchio, P. , Caprio, E. , Nazzi, F. *et al* (2013) Neonicotinoid clothianidin adversely affects insect immunity and promotes replication of a viral pathogen in honey bees. Proceedings of the National Academy of Sciences of the United States of America, 110, 18466–18471. 24145453 10.1073/pnas.1314923110 PMC3831983

[jane12345-bib-0015] Doublet, V. , Labarussias, M. , de Miranda, J.R. , Moritz, R.F.A. & Paxton, R.J. (2014) Bees under stress: sublethal doses of a neonicotinoid pesticide and pathogens interact to elevate honey bee mortality across the life cycle. Environmental Microbiology. doi:10.1111/1462‐2920.12426. 10.1111/1462-2920.12426 25611325

[jane12345-bib-0016] Dray, S. & Dufour, A.B. (2007) The ade4 package: implementing the duality diagram for ecologists. Journal of Statistical Software, 22, 1–20.

[jane12345-bib-0017] Durrer, S. & Schmid‐Hempel, P. (1994) Shared use of flowers leads to horizontal pathogen transmission. Proceedings of the Royal Society Part B Biological Sciences, 258, 299–302.

[jane12345-bib-0018] Evison, S.E. , Roberts, K.E. , Laurenson, L. , Pietravalle, S. , Hui, J. , Biesmeijer, J.C. *et al* (2012) Pervasiveness of parasites in pollinators. PLoS ONE, 7, e30641. 22347356 10.1371/journal.pone.0030641 PMC3273957

[jane12345-bib-0019] Fisher, M.C. , Garner, T.W. & Walker, S.F. (2009) Global emergence of *Batrachochytrium dendrobatidis* and amphibian chytridiomycosis in space, time, and host. Annual Review of Microbiology, 63, 291–310. 10.1146/annurev.micro.091208.073435 19575560

[jane12345-bib-0020] Fitzpatrick, U. , Murray, T.E. , Paxton, R.J. , Breen, J. , Cotton, D. , Santorum, V. *et al* (2007) Rarity and decline in bumblebees ‐ A test of causes and correlates in the Irish fauna. Biological Conservation, 136, 185–194.

[jane12345-bib-0021] Fürst, M.A. , McMahon, D.P. , Osborne, J.L. , Paxton, R.J. & Brown, M.J.F. (2014) Disease associations between honeybees and bumblebees as a threat to wild pollinators. Nature, 506, 364–366. 24553241 10.1038/nature12977 PMC3985068

[jane12345-bib-0022] Genersch, E. & Aubert, M. (2010) Emerging and re‐emerging viruses of the honey bee (*Apis mellifera* L.). Veterinary Research, 41, 54. 20423694 10.1051/vetres/2010027 PMC2883145

[jane12345-bib-0023] Genersch, E. , Yue, C. , Fries, I. & de Miranda, J.R. (2006) Detection of deformed wing virus, a honey bee viral pathogen, in bumble bees (*Bombus terrestris* and *Bombus pascuorum*) with wing deformities. Journal of Invertebrate Pathology, 91, 61–63. 16300785 10.1016/j.jip.2005.10.002

[jane12345-bib-0024] Gill, R.J. , Ramos‐Rodriguez, O. & Raine, N.E. (2012) Combined pesticide exposure severely affects individual‐ and colony‐level traits in bees. Nature, 491, 105–108. 23086150 10.1038/nature11585 PMC3495159

[jane12345-bib-0025] González‐Varo, J.P. , Biesmeijer, J.C. , Bommarco, R. , Potts, S.G. , Schweiger, O. , Smith, H.G. *et al* (2013) Combined effects of global change pressures on animal‐mediated pollination. Trends in Ecology and Evolution, 28, 524–530. 23746938 10.1016/j.tree.2013.05.008

[jane12345-bib-0026] Goulson, D. (2009) Bumblebees. Behaviour, Ecology and Conservation, 2nd edn Oxford University Press, Oxford, UK.

[jane12345-bib-0027] Goulson, D. , Lye, G.C. & Darvill, B. (2008) Decline and conservation of bumble bees. Annual Review of Entomology, 53, 191–208. 10.1146/annurev.ento.53.103106.093454 17803456

[jane12345-bib-0028] Granberg, F. , Vicente‐Rubiano, M. , Rubio‐Guerri, C. , Karlsson, O.E. , Kukielka, D. , Belàk, S. *et al* (2013) Metagenomic detection of viral pathogens in Spanish honeybees: co‐infection by Aphid lethal paralysis virus, Israel acute paralysis and Lake Sinai viruses. PLoS ONE, 8, e57459. 23460860 10.1371/journal.pone.0057459 PMC3583878

[jane12345-bib-0029] Graystock, P. , Yates, K. , Evison, S.E.F. , Darvill, B. , Goulson, D. & Hughes, W.O.H. (2013) The Trojan hives: pollinator pathogens, imported and distributed in bumblebee colonies. Journal of Applied Ecology, 50, 1207–1215.

[jane12345-bib-0030] Klein, A.M. , Vaissiere, B.E. , Cane, J.H. , Steffan‐Dewenter, I. , Cunningham, S.A. , Kremen, C. *et al* (2007) Importance of pollinators in changing landscapes for world crops. Proceedings of the Royal Society Part B Biological Sciences, 274, 303–313. 10.1098/rspb.2006.3721 PMC1702377 17164193

[jane12345-bib-0031] Kosior, A. , Celary, W. , Olejniczak, P. , Fijal, J. , Król, W. , Solarz, W. *et al* (2007) The decline of the bumble bees and cuckoo bees (Hymenoptera: Apidae: Bombini) of Western and Central Europe. Oryx, 41, 79–88.

[jane12345-bib-0032] Levitt, A.L. , Singh, R. , Cox‐Foster, D.L. , Rajotte, E. , Hoover, K. , Ostiquy, N. *et al* (2013) Cross‐species transmission of honey bee viruses in associated arthropods. Virus Research, 176, 232–240. 23845302 10.1016/j.virusres.2013.06.013

[jane12345-bib-0033] Mao, W. , Schuler, M.A. & Berenbaum, M.R. (2013) Honey constituents up‐regulate detoxification and immunity genes in the western honey bee *Apis mellifera* . Proceedings of the National Academy of Sciences of the United States of America, 110, 8842–8846. 23630255 10.1073/pnas.1303884110 PMC3670375

[jane12345-bib-0034] Martin, S.J. , Highfield, A.C. , Brettell, L. , Villalobos, E.M. , Budge, G.E. , Powell, M. *et al* (2012) Global honey bee viral landscape altered by a parasitic mite. Science, 336, 1304–1306. 22679096 10.1126/science.1220941

[jane12345-bib-0035] Mayack, C. & Naug, D. (2009) Energetic stress in the honeybee *Apis mellifera* from *Nosema ceranae* infection. Journal of Invertebrate Pathology, 100, 185–188. 19135448 10.1016/j.jip.2008.12.001

[jane12345-bib-0036] McArt, S.H. , Koch, H. , Irwin, R.E. & Adler, L.S. (2014) Arranging the bouquet of disease: floral traits and the transmission of plant and animal pathogens. Ecology Letters, 17, 624–636. 24528408 10.1111/ele.12257

[jane12345-bib-0037] McMahon, D.P. , Fürst, M.A. , Caspar, J. , Theodorou, J.P. , Brown, M.J.F. & Paxton, R.J. (2015) Data From: A sting in the spit: widespread cross‐infection of multiple RNA viruses across wild and managed bees. Dryad Digital Repository http://dx.doi.org/10.5061/dryad.4b565 10.1111/1365-2656.12345 PMC4832299 25646973

[jane12345-bib-0038] Meeus, I. , Brown, M.J.F. , De Graaf, D.C. & Smagghe, G. (2011) Effects of invasive parasites on bumble bee declines. Conservation Biology, 25, 662–671. 21771075 10.1111/j.1523-1739.2011.01707.x

[jane12345-bib-0039] Meeus, I. , de Miranda, J.R. , De Graaf, D.C. , Wäkers, F. & Smagghe, G. (2014) Effect of oral infection with Kashmir bee virus and Israeli acute paralysis virus on bumblebee (*Bombus terrestris*) reproductive success. Journal of Invertebrate Pathology, 121, 64–69. 25004171 10.1016/j.jip.2014.06.011

[jane12345-bib-0040] de Miranda, J.R. & Genersch, E. (2010) Deformed wing virus. Journal of Invertebrate Pathology, 103, S48–S61. 19909976 10.1016/j.jip.2009.06.012

[jane12345-bib-0041] de Miranda, J.R. , Bailey, L. , Ball, B.V. , Blanchard, P. , Budge, G.E. , Chejanovsky, N. *et al* (2013) Standard methods for virus research in *Apis mellifera* . Journal of Apicultural Research, 52. doi: 10.3896/IBRA.1.52.4.22.

[jane12345-bib-0042] Moore, J. , Jironkin, A. , Chandler, D. , Burroughs, N. , Evans, D.J. & Ryabov, E.V. (2011) Recombinants between Deformed wing virus and *Varroa destructor* virus‐1 may prevail in *Varroa destructor*‐infested honeybee colonies. Journal of General Virololgy, 92, 156–161. 10.1099/vir.0.025965-0 20926636

[jane12345-bib-0043] Peng, W. , Li, J. , Bonchristiani, H. , Strange, J.P. , Hamilton, M. & Chen, Y. (2011) Host range expansion of honey bee black queen cell virus in the bumble bee, *Bombus huntii* . Apidologie, 42, 650–658.

[jane12345-bib-0044] Potts, S.G. , Biesmeijer, J.C. , Kremen, C. , Neumann, P. , Schweiger, O. & Kunin, W.E. (2010) Global pollinator declines: trends, impacts and drivers. Trends in Ecology and Evolution, 25, 345–353. 20188434 10.1016/j.tree.2010.01.007

[jane12345-bib-0045] R Core Team (2013) R: A language and environment for statistical computing. R Foundation for Statistical Computing. http://www.R-project.org (25 November 2013).

[jane12345-bib-0046] Ravoet, J. , De Smet, L. , Meeus, I. , Smagghe, G. , Wenseleers, T. & de Graaf, D.C. (2014) Widespread occurrence of honey bee pathogens in solitary bees. Journal of Invertebrate Pathology, 122, 55–58. 25196470 10.1016/j.jip.2014.08.007

[jane12345-bib-0047] Rosenkranz, P. , Aumeier, P. & Ziegelmann, B. (2010) Biology and control of *Varroa destructor* . Journal of Invertebrate Pathology, 103, S96–S119. 19909970 10.1016/j.jip.2009.07.016

[jane12345-bib-0048] Ruiz‐González, M.X. , Bryden, J. , Moret, Y. , Reber Funk, C. , Schmid‐Hempel, P. & Brown, M.J.F. (2012) Dynamic transmission, host quality, and population structure in a multihost parasite of bumblebees. Evolution, 66, 3053–3066. 23025597 10.1111/j.1558-5646.2012.01655.x

[jane12345-bib-0049] Runckel, C. , Flenniken, M.L. , Engel, J.C. , Ruby, J.G. , Ganem, D. , Andino, R. *et al* (2011) Temporal analysis of the honey bee microbiome reveals four novel viruses and seasonal prevalence of known viruses, Nosema and Crithidia. PLoS ONE, 6, e20656. 21687739 10.1371/journal.pone.0020656 PMC3110205

[jane12345-bib-0050] Santillán‐Galicia, M.T. , Ball, B.V. , Clark, S.J. & Alderson, P.G. (2014) Slow bee paralysis virus and its transmission in honey bee pupae by *Varroa destructor* . Journal of Apicultural Research, 53, 146–154.

[jane12345-bib-0051] Schmid‐Hempel, R. , Eckhardt, M. , Goulson, D. , Heinzmann, D. , Lange, C. , Plischuk, S. *et al* (2014) The invasion of southern South America by imported bumblebees and associated parasites. Journal of Animal Ecology, 83, 823–837. 24256429 10.1111/1365-2656.12185

[jane12345-bib-0052] Schroeder, D.C. & Martin, S.J. (2012) Deformed wing virus: the main suspect in unexplained honeybee deaths worldwide. Virulence, 3, 589–591. 23154287 10.4161/viru.22219 PMC3545936

[jane12345-bib-0053] Singh, R. , Levitt, A.L. , Rajotte, E.G. , Holmes, E.C. , Ostiguy, N. , Vanengelsdorp, O. *et al* (2010) RNA viruses in hymenopteran pollinators: evidence of inter‐taxa virus transmission via pollen and potential impact on non‐*Apis* hymenopteran species. PLoS ONE, 5, e14357. 21203504 10.1371/journal.pone.0014357 PMC3008715

[jane12345-bib-0054] Sumpter, D.J.T. & Martin, S.J. (2004) The dynamics of virus epidemics in *Varroa*‐infested honey bee colonies. Journal of Animal Ecology, 73, 51–63.

[jane12345-bib-0055] Vanbergen, A.J. , Baude, M. , Biesmeijer, J.C. , Britton, N.F. , Brown, M.J.F. , Bryden, J. *et al* (2013) Threats to an ecosystem service: pressures on pollinators. Frontiers in Ecology and the Environment, 11, 251–259.

[jane12345-bib-0056] Whitehorn, P.R. , O'Connor, S. , Wackers, F.L. & Goulson, D. (2012) Neonicotinoid pesticide reduces bumble bee colony growth and queen production. Science, 336, 351–352. 22461500 10.1126/science.1215025

[jane12345-bib-0057] Williams, P.H. (1982) The distribution and decline of British bumble bees (*Bombus* Latr.). Journal of Apicultural Research, 21, 236–245.

[jane12345-bib-0058] Williams, P.H. & Osborne, J.L. (2009) Bumblebee vulnerability and conservation world‐wide. Apidologie, 40, 367–387.

[jane12345-bib-0059] Williamson, S.M. & Wright, G.A. (2013) Exposure to multiple cholinergic pesticides impairs olfactory learning and memory in honeybees. Journal of Experimental Biology, 216, 1799–1807. 23393272 10.1242/jeb.083931 PMC3641805

[jane12345-bib-0060] Woolhouse, M.E.J. , Haydon, D.T. & Antia, R. (2005) Emerging pathogens: the epidemiology and evolution of species jumps. Trends in Ecology and Evolution, 20, 238–244. 16701375 10.1016/j.tree.2005.02.009 PMC7119200

[jane12345-bib-0061] Zuur, A.F. , Ieno, E.N. , Walker, N.J. , Saveliev, A.A. & Smith, G.M. (2009). Mixed effects models and extensions in ecology with R. New York, Springer.

