# Supplementary material for: A sting in the spit: widespread cross‐infection of multiple RNA viruses across wild and managed bees
Source: J Anim Ecol. 2015 Mar 3;84(3):615–24. doi: 10.1111/1365-2656.12345 (PMC4832299; doi:10.1111/1365-2656.12345)
Supplement: Supplementary file 1 — Table S1. List of qRT‐PCR primers used in this study [file JANE-84-615-s001.pdf]

Primer Target	Name	Sequence	Application	Reference
VDV (RdRp)	VDV-F2	TATCTTCATTAAAACCGCCAGGCT	qPCR	This study
VDV (RdRp)	VDV-R2a	CTTCCTCATTAACTGAGTTGTTGTC	qPCR	This study
DWV (RdRp)	DWV-F2	TGTCTTCATTAAAGCCACCTGGAA	qPCR	This study
DWV (RdRp)	DWV-R2a	TTTCCTCATTAACTGTGTCGTTGAT	qPCR	This study
DWV (RdRp)	DWV-F1a	GGAAACATCTGGAATTAGCGACAAA	Stand' curve	This study
VDV (RdRp)	VDV-F1a	GAAAACATTTGGAATTAGCAACGAC	Stand' curve	This study
DWV/VDV (RdRp)	DWVDV-7A-R	AATCCGTGAATATAGTGTGAGG	Stand' curve	This study
SBPV	SPV-F3177	GCGCTTTAGTTCAATTGCC	qPCR	[1]
SBPV	SPV-B3363	ATTATAGGACGTGAAAATATAC	qPCR	"
ABPV	ABPV-F6548	TCATACCTGCCGATCAAG	qPCR	[2]
KBV	KBV-F6639	CCATACCTGCTGATAACC	qPCR	
IAPV	IAPV-F6627	CCATGCCTGGCGATTCAC	qPCR	"
ABPV/IAPV	KIABPV-B6707	CTGAATAATACTGTGCGTATC	qPCR	"
BQCV	BQCV-F7893	AGTGGCGGAGATGTATGC	qPCR	[3]
BQCV	BQCV-B8150	GGAGGTGAAGTGGCTATATC	qPCR	"
SBV	SBV-F3164	TTGGAACTACGCATTCTCTG	qPCR	"
SBV	SBV-B3461	GCTCTAACCTCGCATCAAC	qPCR	"
β-actin	Am-actin2-qF	CGTGCCGATAGTATTCTTG	qPCR	"
β-actin	Am-actin2-qB	CTTCGTCACCAACATAGG	qPCR	"

Table S1. List of RT-PCR primers used in this study alongside their use and reference.

1. de Miranda, J.R. *et al.* (2010). Genetic characterization of slow bee paralysis virus of the honeybee (*Apis mellifera* L.). *J. Gen. Virol.,* 91, 2524-2530.
2. de Miranda, J.R., Cordoni, G., & Budge, G. (2010). The Acute bee paralysis virus-Kashmir bee virus-Israeli acute paralysis virus complex. *J. Invertebr. Pathol.,* 103, S30-47 (2010)
3. Locke, B., Forsgren, E., Fries, I., & de Miranda, J.R. (2012). Acaricide treatment affects viral dynamics in *Varroa destructor*-infested honey bee colonies via both host physiology and mite control. *Appl. Environ. Microb.,* 78, 227-235.
