# Supplementary material for: A sting in the spit: widespread cross‐infection of multiple RNA viruses across wild and managed bees
Source: J Anim Ecol. 2015 Mar 3;84(3):615–24. doi: 10.1111/1365-2656.12345 (PMC4832299; doi:10.1111/1365-2656.12345)
Supplement: Supplementary file 2 — Table S2. Detailed sample information by collection site [file JANE-84-615-s002.pdf]

Site	Collection Date	Honeybees	Bumblebees	Species 1 (N)	Species 2 (N)	Species 3 (N)	Species 4 (N)
A	7/22/11	10	24	*B. pascuorum* (10)	*B. terrestris* (7)	*B. lucorum* (7)	
B	8/14/11	10	19	*B. hortorum* (10)	*B. lucorum* (9)		
C	8/2/11	9	20	*B. pascuorum* (10)	*B. lucorum* (7)	*B. terrestris* (3)	
D	8/16/11	10	16	*B. lapidarius* (10)	*B. lucorum* (4)	*B. terrestris* (2)	
E	7/22/11	10	15	*B. lapidarius* (10)	*B. lucorum* (2)	*B. terrestris* (2)	*B.* sp. (1)**
F	7/24/11	7	13	*B. lapidarius* (9)	*B. terrestris* (4)		
G**	7/20/11	0	15	*B. lapidarius* (10)	*B. lucorum* (4)	*B. terrestris* (3)	
H	7/31/11	10	12	*B. lapidarius* (7)	*B. lucorum* (4)	*B. terrestris* (1)	
I	6/24/11	4	11	*B. lapidarius* (6)	*B. lucorum* (5)		
J	6/16/11	8	19	*B. lapidarius* (11)	*B. lucorum* (4)	*B. terrestris* (4)	
K	6/15/11	4	16	*B. lapidarius* (10)	*B. lucorum* (4)	*B. terrestris* (2)	
L	6/26/11	10	19	*B. lapidarius* (9)	*B. hortorum* (6)	*B. terrestris* (3)	*B. lucorum* (1)
M	6/5/11	17	27	*B. lapidarius* (15)	*B. terrestris* (8)	*B. hortorum* (3)	*B. pratorum* (1)
N	6/25/11	10	12	*B. lapidarius* (6)	*B. terrestris* (4)	*B. lucorum* (2)	
O	7/25/11	8	17	*B. pascuorum* (10)	*B. terrestris* (5)	*B. hortorum* (1)	*B.* sp. (1)
P	7/28/11	10	17	*B. monticola* (7)	*B. terrestris* (5)	*B.* spp. (5)**	
Q	6/27/11	9	19	*B. lapidarius* (10)	*B. terrestris* (7)	*B. lucorum* (2)	
R	6/24/11	10	9	*B. lapidarius* (4)	*B. terrestris* (2)	*B. lucorum* (2)	*B. jonellus* (1)
S	6/30/11	10	20	*B. lapidarius* (9)	*B. lucorum* (6)	*B. terrestris* (3)	*B. pratorum* (2)
T	7/9/11	15	28	*B. lapidarius* (15)	*B. terrestris* (9)	*B. lucorum* (4)	
U	7/23/11	10	20	*B. pascuorum* (10)	*B. terrestris* (6)	*B. lucorum* (4)	
V	7/3/11	9	14	*B. lapidarius* (9)	*B. terrestris* (3)	*B. lucorum* (2)	
W	6/15/11	9	18	*B. lapidarius* (9)	*B. lucorum* (8)	*B. terrestris* (1)	
X	6/14/11	9	20	*B. lapidarius* (10)	*B. lucorum* (7)	*B. terrestris* (3)	
Y	7/29/11	10	20	*B. hortorum* (10)	*B. pascuorum* (10)		
Z	7/9/11	9	13	*B. terrestris* (5)	*B. pascuorum* (5)	*B. lucorum* (3)	

Table S2. Total sample size and species composition (passing quality control) at each site. Unidentified species given as '*B.* sp.' **Not used in GLMM analyses.
