# Supplementary material for: A sting in the spit: widespread cross‐infection of multiple RNA viruses across wild and managed bees
Source: J Anim Ecol. 2015 Mar 3;84(3):615–24. doi: 10.1111/1365-2656.12345 (PMC4832299; doi:10.1111/1365-2656.12345)
Supplement: Supplementary file 3 — Table S3. GLMM and GLM final models with A. mellifera virus prevalence as the response variable [file JANE-84-615-s003.pdf]

a) GLMM						
Response	Virus	Parameters	Estimate	SE	z-value	*P*-value
Apis virus prevalence	BQCV	Intercept	-2.505	0.338	-7.421	
		Bombus BQCV	0.890	0.288	3.091	0.002*
	DWV	Intercept	-0.843	0.287	-2.934	
		Bombus DWV	0.485	0.280	1.731	0.083
	ABPV	Intercept	-5.242	1.112	-4.713	
		Bombus ABPV	1.873	0.670	2.796	0.005*
b) GLM						
Apis virus prevalence		Intercept	0.299	0.078	3.831	
		Bombus all viruses	1.002	0.220	4.546	<0.001*
		Latitude	-0.100	0.057	-1.736	0.097

Table S3. (a) Best GLMM models explaining individual virus prevalence in *A. mellifera* using AIC for model selection. *Significant variables. $r^2_c = 0.36; 0.36; 0.64$ and $r^2_m = 0.15; 0.19; 0.38$ for BQCV, DWV and ABPV, respectively. (b) Best GLM model explaining total RNA virus prevalence in *A. mellifera*, using AICc for model selection (b). *Significant variables. Nagelkerke $r^2 = 0.59$
