# Supplementary material for: A sting in the spit: widespread cross‐infection of multiple RNA viruses across wild and managed bees
Source: J Anim Ecol. 2015 Mar 3;84(3):615–24. doi: 10.1111/1365-2656.12345 (PMC4832299; doi:10.1111/1365-2656.12345)
Supplement: Supplementary file 4 — Fig. S1. BQCV, ABPV and SBPV cloned sequence alignments. [file JANE-84-615-s004.pdf]

(a)BQCV

```
Ref. NC_003784     TAGTGGCGGAGATGTATGCGCTTTATCGAGGAGGAGTTCGAGTTAAAGTAGTTACTGAGAAGGGTGTAGATTTCGTCAGAGCTACCGTTAG
A.mellifera  H 1  .................................................T.................G.......................
A.mellifera  H 2  .................................................T.................G.......................
A.mellifera  H 3  .................................................T..............C..G.......................
A.mellifera  H 4  .................................................T.................G.......................
A.mellifera  H 5  .................................................T.................G.......................
B.lucorum    K 1  .................................................T.................G.................T.....
B.lucorum    K 2  .................................................T.................G.................T.....
B.lucorum    K 3  .................................................T.................G.................T.....
B.lucorum    K 4  .................................................T.................G.................T.....
B.terrestris N 1  NNN..............................................T.................G.......................
B.terrestris N 2  .................................................T.................G.......................
B.terrestris N 3  ...................................................................G....................G..
B.terrestris N 4  ...................................................................G....................G..

Ref. NC_003784     TCCTCAACAGACTTATGGTAGCGATGTCGCTCCTACTACTCACATCAGTACTCCTTTGGCAATAGAACAAATACCTATAAAGGGAGTCGCAGAGTTC
A.mellifera  H 1  ..................C..T....................T......................................................
A.mellifera  H 2  ..................C..T....................T......................................................
A.mellifera  H 3  ..................C..T....................T......................................................
A.mellifera  H 4  ..................C..T....................T......................................................
A.mellifera  H 5  ..................C..T....................T......................................................
B.lucorum    K 1  ......G...........C..T....................T......................................................
B.lucorum    K 2  ..................C..T....................T......................................................
B.lucorum    K 3  ......G...........C..T....................T......................................................
B.lucorum    K 4  ..................C..T....................T......................................................
B.terrestris N 1  ...............C..C..T..A.................T......................................................
B.terrestris N 2  ...............C..C..T..A.................T......................................................
B.terrestris N 3  .....................T.................C........C..........................C.....................
B.terrestris N 4  .....................T.................C........C..........................C.....................

Ref. NC_003784     CAAATACCGTACTATGCTCCATGTTTGTCATCTTCGTTTAGAGCGAATTCGGAAACATTTTACTATAGTTCAGGTCGGAATAATCTCGATATAGCCACTTCACCTCC
A.mellifera  H 1  ...........................................................C..T............................................
A.mellifera  H 2  ...........................................................C..T............................................
A.mellifera  H 3  ...........................................................C..T............................................
A.mellifera  H 4  ...........................................................C..T............................................
A.mellifera  H 5  ...........................................................C..T....................................NNNNNNNN
B.lucorum    K 1  ...........................................................C...............................................
B.lucorum    K 2  ...........................................................C...............................................
B.lucorum    K 3  ...........................................................C...............................................
B.lucorum    K 4  ...........................................................C...............................................
B.terrestris N 1  ...........................................................................................................
B.terrestris N 2  ......................................C....................................................................
B.terrestris N 3  ....................G.....A.......................A........................................................
B.terrestris N 4  ....................G.....A.......................A........................................................
```

(b)ABPV

```
Ref. NC_002548     GATCAAGAAACAAATACTTCCAACGTACATAATACGCAACTCGCGTCGACCTCTGAAGAAAACTCAGTTGAAACGGAACAAATCACCACCTT
A.mellifera  M 1   ............................................................................................
A.mellifera  M 2   ............................................................................................
A.mellifera  M 3   ............................................................................................
A.mellifera  M 4   NNNNNNNNNNNNNNNNNNNNNNNNNNNNNN..............................................................
B.terrestris M 1   ............................................................................................
B.terrestris M 2   ............................................................................................
B.terrestris M 3   ............................................................................................

Ref. NC_002548     TCATGATGTGGAAACTCCAAATAGGATCAATACCCCCATGGCTCAAGACACTTCATCGGCTCGGAGCATGGATGATACGCACAGTATTATTCAG
A.mellifera  M 1   ............................G...................T.............................................
A.mellifera  M 2   ............................G...................T...............................NNNNNNNNNNNNNN
A.mellifera  M 3   ............................G...................T.............................................
A.mellifera  M 4   ............................G...................T.............................................
B.terrestris M 1   ............................G...................T.............................................
B.terrestris M 2   ............................G...................T.............................................
B.terrestris M 3   ............................G...................T.............................................
```

(c) SBPV

```
Ref. NC_014137     GCGCTTTAGTTCAATTGCCTCTCTATCCCCATGGATTAAGAATACAGCCTGACGTTAATAATCCAATATGGAATATTATGCGTGATGGTCATATTCCTGTTATTTCAAGTGGA
A.mellifera   C 1  ..............C..................................................................................................
A.mellifera   C 2  .................................................................................................................
A.mellifera   C 3  .................................................................................................................
A.mellifera   C 4  .................................................................................................................
A.mellifera   C 5  .................................................................................................................
B.pascuorum   C 1  .................................................................................................................
B.pascuorum   C 2  .................................................................................................................
B.pascuorum   C 3  .................................................................................................................
B.pascuorum   C 4  .................................................................................................................
B.pascuorum   C 5  .................................................................................................................
B.terrestris C 1  ...................C.............................................................................................
B.terrestris C 2  ...................C.............................................................................................
B.terrestris C 3  ...................C.............................................................................................
B.terrestris C 4  ...................C.............................................................................................
B.terrestris C 5  ...................C.............................................................................................

Ref. NC_014137     TTCAGGTATTTTAGAGGAGGATTACGACTCCGCATAGTTGTTGAAGGTCTTAATAGCTGTGTTTGGGTGCAACATCATCCTGATAGACCTAGTATATTTTCACGTCCTATAAT
A.mellifera   C 1  .................................................................................................................
A.mellifera   C 2  .................................................................................................................
A.mellifera   C 3  .................................................................................................................
A.mellifera   C 4  .................................................................................................................
A.mellifera   C 5  .................................................................................................................
B.pascuorum   C 1  .................................................................................................................
B.pascuorum   C 2  .................................................................................................................
B.pascuorum   C 3  ................................................T................................................................
B.pascuorum   C 4  .................................................................................................................
B.pascuorum   C 5  .................................................................................................................
B.terrestris C 1  .......................................................................G.........................................
B.terrestris C 2  .......................................................................G.........................................
B.terrestris C 3  .......................................................................G.........................................
B.terrestris C 4  ........................................C..............................G.........................................
B.terrestris C 5  ........................................C..............................G.........................................
```

Fig. S1. (a) BQCV partial sequence alignment of up to four clones from a single forager of *A. mellifera*, *B. lucorum* and *B. terrestris* from sites H, K, and N respectively. (b) ABPV partial sequence alignment of up to four clones from a single *A. mellifera* and *B. terrestris* forager from site M. (c) SBPV partial sequence alignment of up to five clones from single foragers of *A. mellifera*, *B. pascuorum*, and *B. terrestris* from site C.
