# Supplementary material for: A sting in the spit: widespread cross‐infection of multiple RNA viruses across wild and managed bees
Source: J Anim Ecol. 2015 Mar 3;84(3):615–24. doi: 10.1111/1365-2656.12345 (PMC4832299; doi:10.1111/1365-2656.12345)
Supplement: Supplementary file 5 — Fig. S2. Phylogenetic trees of cloned BQCV and SBPV sequences. [file JANE-84-615-s005.pdf]

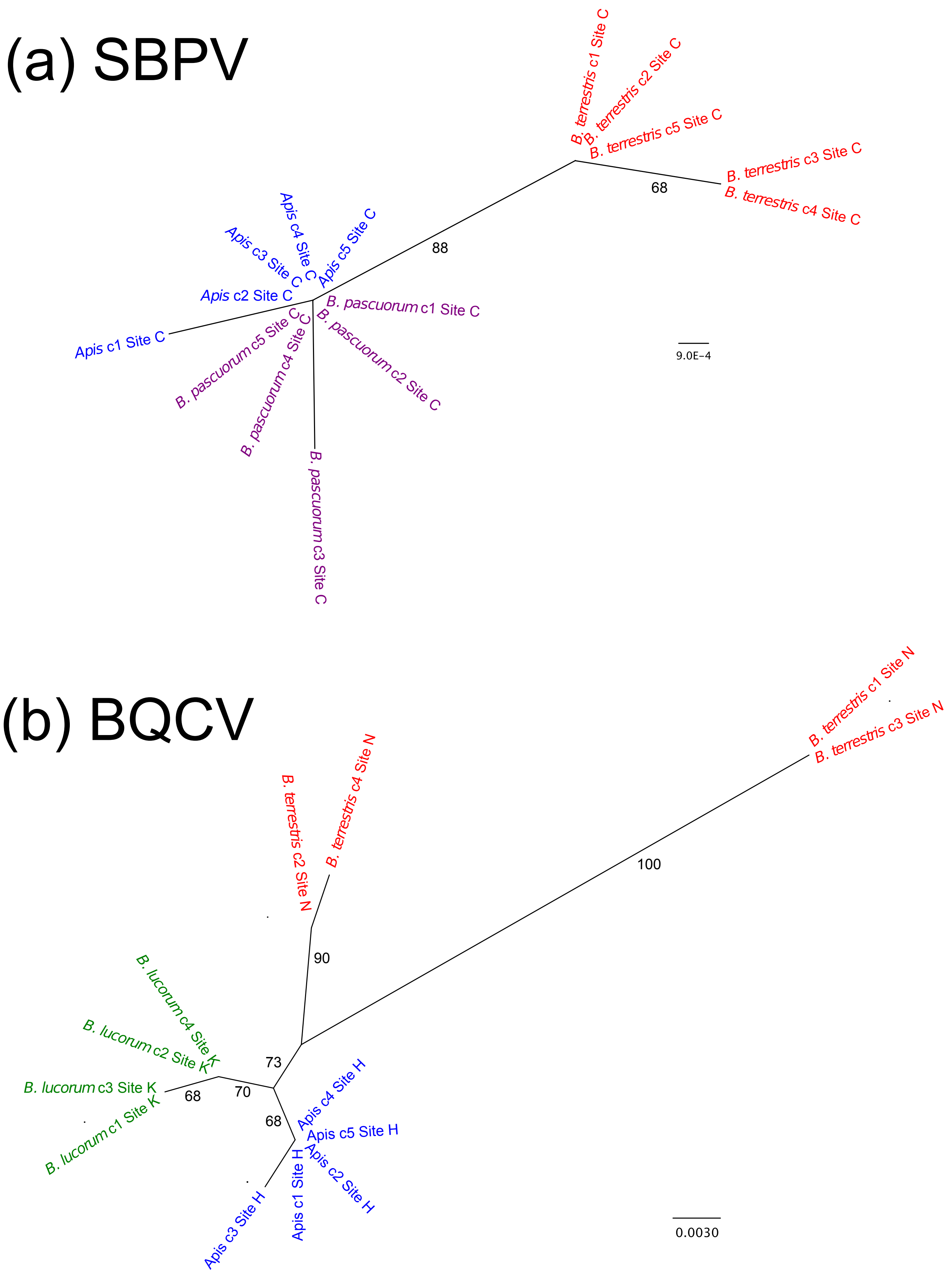

Fig. S2. Unrooted Maximum Likelihood trees (General Time Reversible model, uniform rates) of sequenced viral clones. Percent bootstrap support is indicated at relevant nodes (500 replicates). Coloured tip labels are cloned sequences from individual hosts, with each colour representing a different species. Clone number (c1-5) and site information are also given. (a) SBPV: (b) BQCV
