# Supplementary material for: A sting in the spit: widespread cross‐infection of multiple RNA viruses across wild and managed bees
Source: J Anim Ecol. 2015 Mar 3;84(3):615–24. doi: 10.1111/1365-2656.12345 (PMC4832299; doi:10.1111/1365-2656.12345)
Supplement: Supplementary file 6 — Fig. S3. Summary prevalence (%) of RNA viruses in A. mellifera and Bombus spp. [file JANE-84-615-s006.pdf]

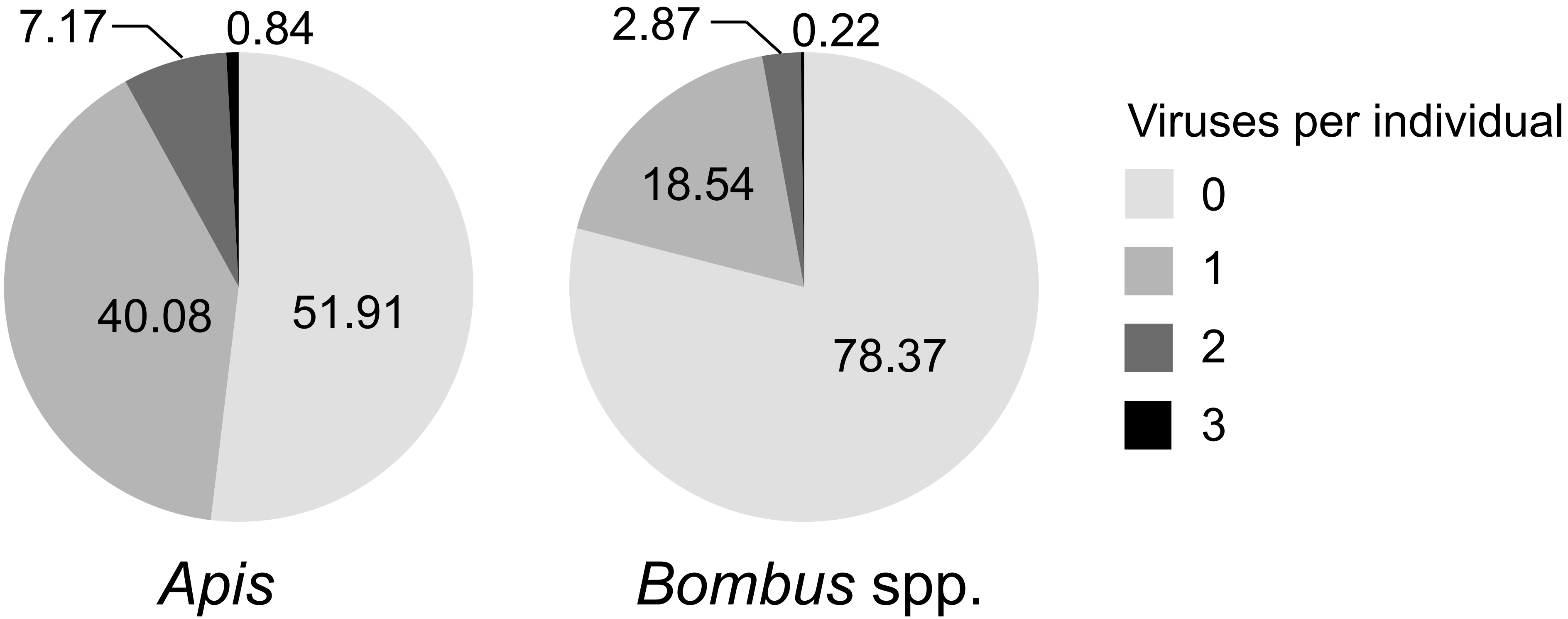

Fig. S3. Summary prevalence (%) of RNA viruses in *A. mellifera* and *Bombus* spp. pollinators. Note that values indicated are based on the raw data and not true prevalences
