# Supplementary material for: A sting in the spit: widespread cross‐infection of multiple RNA viruses across wild and managed bees
Source: J Anim Ecol. 2015 Mar 3;84(3):615–24. doi: 10.1111/1365-2656.12345 (PMC4832299; doi:10.1111/1365-2656.12345)
Supplement: Supplementary file 7 — Fig. S4. Linear regressions of raw A. mellifera and Bombus spp. RNA virus prevalence data. [file JANE-84-615-s007.pdf]

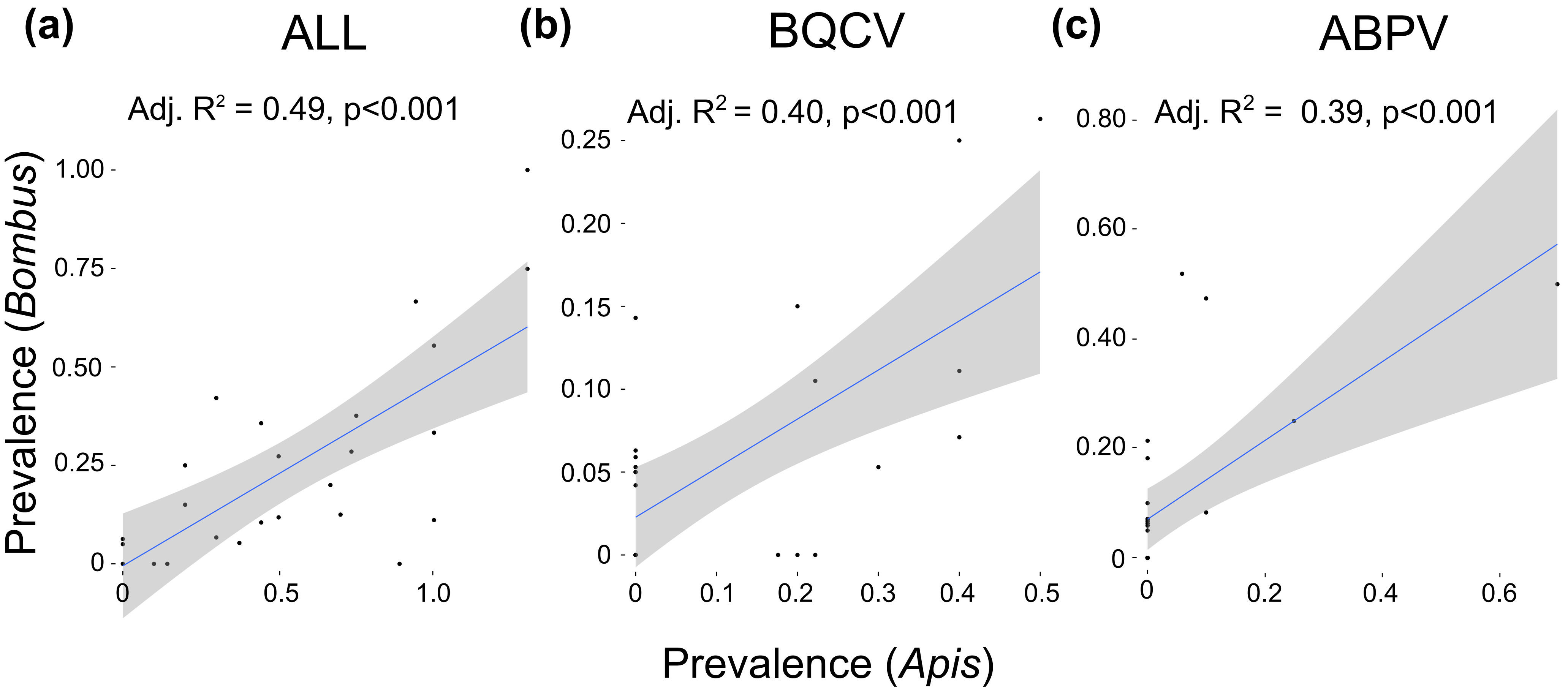

Fig. S4. Linear regression of raw data illustrating the relationship between virus prevalence in *Bombus* spp. and *A. mellifera*: (a) All viruses combined (Adj. R^2 = 0.49, p<0.001); (b) BQCV (Adj. R^2 = 0.40, p<0.001); (c) ABPV (Adj. R^2 = 0.39, p<0.001).
